# Differential control of Toll-like receptor 4–induced interleukin-10 induction in macrophages and B cells reveals a role for p90 ribosomal S6 kinases

**DOI:** 10.1074/jbc.M117.805424

**Published:** 2017-12-11

**Authors:** Ruhcha V. Sutavani, Iain R. Phair, Rebecca Barker, Alison McFarlane, Natalia Shpiro, Stuart Lang, Andrew Woodland, J. Simon C. Arthur

**Affiliations:** From the ‡ Division of Cell Signalling and Immunology,; the § MRC Protein Phosphorylation and Ubiquitylation Unit, and; the ¶ Drug Discovery Unit, Division of Biological Chemistry and Drug Discovery, School of Life Sciences, University of Dundee, Dundee DD1 5EH, Scotland, United Kingdom

**Keywords:** cAMP response element-binding protein (CREB), extracellular-signal-regulated kinase (ERK), p38 MAPK, RSK, Toll-like receptor 4 (TLR4), Interleukin 10, MSK1

## Abstract

Increasing evidence has linked dysregulated interleukin (IL)-10 production by IL-10^+ve^ B cells to autoimmunity, highlighting the importance of improving the understanding of the regulation of IL-10 production in these cells. In both B cells and myeloid cells, IL-10 can be produced in response to Toll-like receptor (TLR) agonists. In macrophages, previous studies have established that mitogen- and stress-activated protein kinases (MSKs) regulate IL-10 production via the phosphorylation of cAMP response element–binding (CREB) protein on the IL-10 promoter. We found here that although MSKs are activated in peritoneal B cells in response to TLR4 agonists, neither MSKs nor CREB are required for IL-10 production in these cells. Using a combination of chemical inhibitors and knockout mice, we found that IL-10 induction in B cells was regulated by an ERK1/2- and p90 ribosomal S6 kinase-dependent mechanism, unlike in macrophages in which p90 ribosomal S6 kinase was not required. This observation highlights fundamental differences in the signaling controlling IL-10 production in B cells and macrophages, even though these two cell types respond to a common TLR stimulus.

## Introduction

IL-10^2^ is an important anti-inflammatory cytokine that plays important roles in limiting inflammation, as evidenced by the development of inflammatory bowel disorders, in both mice and humans, following the loss of IL-10 function (1–3). IL-10 can be produced by a number of cell types, including myeloid cells, T cells, and B cells, in response to a range of different stimuli (reviewed in Refs. 4–6). *In vivo* IL-10 has multiple roles; however, a major component of its anti-inflammatory function is to repress the production of pro-inflammatory cytokines by macrophages and dendritic cells (4–6). Despite its strong anti-inflammatory properties, recombinant IL-10 has not proven to be successful for the treatment of autoimmune disorders (7). This suggests that the timing and location of IL-10 production and/or action are critical for its protective effects. Support for this idea has come from the use of conditional IL-10 knockout mice. Loss of IL-10 specifically in the T-cell compartment was sufficient to promote the development of colitis, whereas myeloid-specific IL-10 deletion did not result in the development of colitis but did sensitize mice to LPS-mediated endotoxic shock (8, 9). Furthermore, transfer of IL-10–competent immune cells can be protective in autoimmune models in mice. For example, transfer of B cells with the potential to make IL-10 has been found to be protective in mouse models of arthritis, autoimmune encephalomyelitis lupus, and colitis (10–15). Although initially described in mice, IL-10–producing B cells have now been identified and have been found to be decreased in several autoimmune conditions including lupus, rheumatoid arthritis, psoriasis, and multiple sclerosis (reviewed in Ref. 16).

The molecular mechanisms behind the regulation of IL-10 production have been studied mainly in T cells and macrophages and differences exist between these cell types in terms of the stimuli and transcription factors that regulate IL-10 transcription (reviewed in Refs. 4–6). In both myeloid and B cells, the activation of pattern recognition receptors, notably members of the Toll-like receptor (TLR) family, have been found to be effective stimuli for inducing IL-10 production (17–19). Much of our understanding about how TLRs drive IL-10 production has come from studies on macrophages and dendritic cells. In these cells, stimulation of TLRs results in the transcriptional activation of the IL-10 gene, thereby giving rise to increased IL-10 protein production and secretion. TLRs activate the MAPK and NFκB pathways, and inhibition of these pathways can prevent TLR-induced cytokine production (20, 21). In the context of IL-10, the ERK1/2 and p38 MAPK pathways have been shown to be important for the control of IL-10 production in macrophages (22). Both ERK1/2 and p38 are able to activate downstream kinases; p38α activates the related kinases MK2 and MK3, whereas ERK1/2 is able to activate RSK1, 2, and 3 (23). p38α and ERK1/2 are both able to activate MSK1 and 2 and for stimuli, such as TLR agonists, that activate both ERK1/2 and p38α; inhibition of both pathways is required to prevent MSK activation (24). Although the role of RSK in IL-10 induction has not been addressed, roles for both MSK1/2 and MK2/3 have been identified in macrophages. MK2 has been reported to reduce IL-10 production by LPS–stimulated bone marrow–derived macrophages (BMDMs) (25). MK2 is known to phosphorylate proteins such as TTP that regulate the stability of cytokine mRNAs (26). Consistent with this, MK2 knockout decreased IL-10 mRNA stability (25). Double knockout of MSK1 and 2 impairs IL-10 production in both BMDMs and dendritic cells (27–29). In this context MSKs activate the transcription factor CREB by phosphorylating it on Ser^133^, resulting in the induction of CREB–dependent genes (30). Similar to MSK1/2 knockouts, BMDMs from mice with a Ser^133^ to Ala knockin mutation in CREB show decreased IL-10 transcription in response to LPS (27). The importance of CREB for the induction of IL-10 in macrophages has been further illustrated by the identification of CREB–binding sites in the IL-10 promoter (22, 31, 32). Furthermore, PGE_2_ can synergistically activate IL-10 transcription in combination with LPS, a process that requires the nuclear translocation of a CREB co-activator protein, CRTC3 (33, 34).

In mice, B cells can be divided into B1 and B2 lineages (35). Most B2 cell subsets do not appear to make appreciable amounts of IL-10 following stimulation. Instead the production of IL-10 is limited to specific subsets that have been termed B10 or regulatory B cells (reviewed in Refs. 36–39). Adoptive transfer of these cells has been shown to be protective in multiple immune models in mice. In addition to specific B2 cell subsets, some B1 cells also have the ability to make IL-10. At present, a master transcription factor has not been identified for the development of IL-10–producing B cells, nor has a unique cell surface maker been found to allow their easy identification. It is possible that IL-10–producing B cells do not represent a unique lineage, with several B-cell subsets having the ability to secrete IL-10 under specific circumstances (36–39).

In mice, the peritoneal cavity is a rich source of IL-10 competent B cells. Unlike splenic B cells, in which only 1–2% of B cell make IL-10, between 20 and 40% of peritoneal cavity B cells are able to produce IL-10 in response to TLR stimulation (39, 40). Little is known about the intracellular pathways controlling IL-10 production in B cells. Here, we show that B cells and macrophages regulate IL-10 production by distinct mechanisms in response to TLR stimulation.

## Results

### Peritoneal cavity B cells and macrophages produce IL-10 in response to ex vivo TLR stimulation

**Figure 1.**
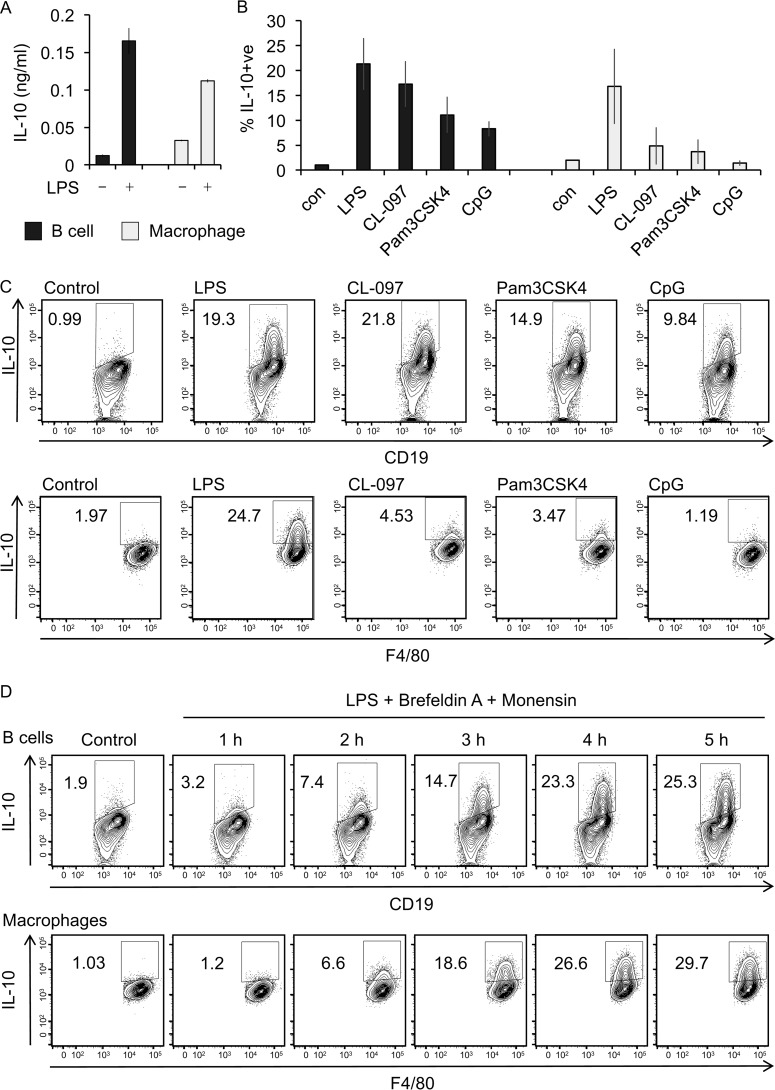
**TLR stimulation induced IL-10 production by peritoneal cavity B cells and macrophages.** *A*, cells were isolated from the peritoneal cavity of wildtype mice and FACS sorted into pure populations of B cells and macrophages as described under “Materials and methods.” The cells were either left unstimulated (control) or were stimulated with 10 μg/ml LPS for 16 h. Culture supernatant was harvested, and concentration of secreted IL-10 was determined by a Luminex assay. The data shown are means ± standard deviation of biological quadruplicates (*n* = 4). *B*, peritoneal cavity cells were stimulated *ex vivo* with 10 μg/ml LPS, 1 μg/ml Pam3CSK, 5 μg/ml CL-097, or 4 μg/ml CpG in the presence of Golgi inhibitors, 3 μg/ml brefeldin A, and 2 μm monensin for 5 h. Cells treated with 3 μg/ml brefeldin A and 2 μm monensin but without LPS stimulation were used as controls (*con*). Subsequently, the cells were stained for lineage markers, CD19 (B cells) and F4/80 (macrophages), and for intracellular IL-10, as described under Materials and methods.” The graphs represent the means and standard deviation for the percentage of IL-10+ve cells in the B-cell and macrophage gates as assessed by flow cytometry (*n* = 3). *C*, representative FACS plots from *B* are shown. *D*, peritoneal cells from wildtype mice were treated with 3 μg/ml brefeldin A, 2 μm monensin, and 10 μg/ml LPS for the indicated times. The level of intracellular IL-10 in the B-cell macrophage populations was determined by flow cytometry.

In unchallenged mice, the peritoneal cavity contains a number of immune cells types with a high proportion of these cells being macrophages or B cells, both of which have the potential to produce cytokines in response to TLR agonists. To determine whether both peritoneal macrophages and B cells produce IL-10, peritoneal cells were sorted into pure (<98%) populations of B cells (CD19+ve cells) and macrophages (F4/80+ve cells) by FACS. The cells were then LPS–stimulated and secretion of IL-10 measured. Both purified macrophages and B cells secreted IL-10 in response to LPS (Fig. 1*A*). IL-10 induction after *ex vivo* LPS stimulation of unsorted peritoneal cells was also assessed using a flow cytometry assay as described under “Materials and methods.” The cells were simultaneously stained for lineage markers for B cells (CD19) and macrophages (F4/80) and intracellular IL-10. In this assay, both B cells (CD19+ve) and macrophages (F4/80+ve) produced IL-10 following LPS stimulation (Fig. 1, *B* and *C*). The ability of B cells to induce IL-10 was not restricted to TLR4 agonists, because CpG (TLR9 agonist), Pam3CSK4 (TLR1/2 agonist), and CL097 (TLR7/8 agonist) were also able to induce IL-10 in peritoneal B cells (Fig. 1, *B* and *C*). Intracellular staining for IL-10 showed that some IL-10 induction could be seen as early as 2 h after LPS stimulation, with levels increasing with time and starting to plateau at 5 h after LPS stimulation (Fig. 1*D*). Peritoneal B cells are a mix of different subsets. Simultaneous analysis of cell surface markers and IL-10 production in LPS–stimulated B cells showed that most of the IL-10–producing B cells were CD19^hi^ and expressed high levels of CD1d, CD5, and IgM but low levels of IgD (Fig. S1), consistent with the cells belonging to the B1a subset.

**Figure 2.**
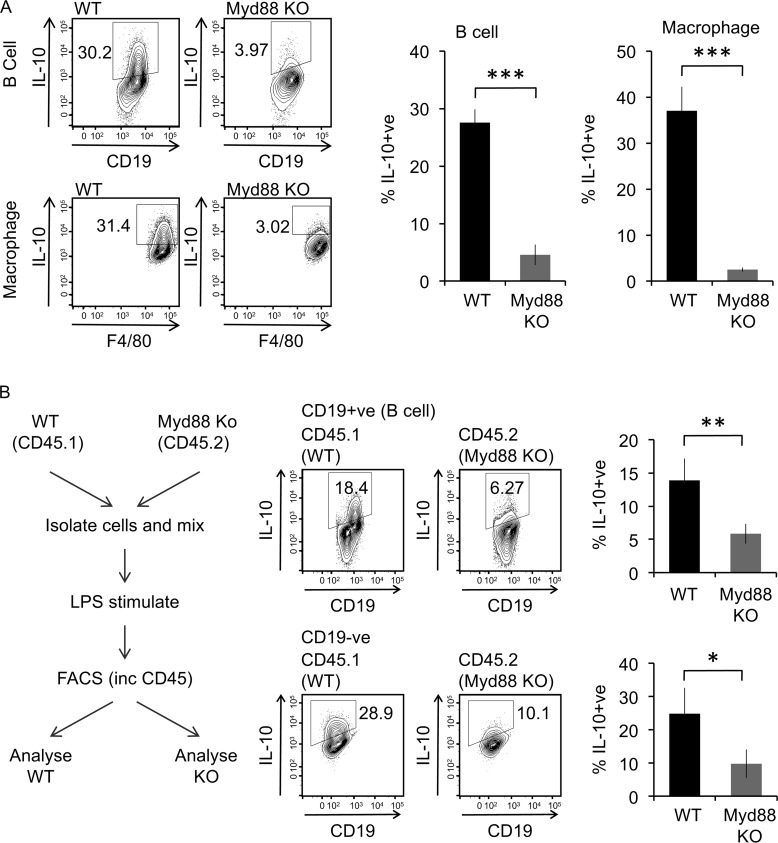
**Effect of Myd88 knockout on IL-10 induction by LPS.** *A*, peritoneal cavity cells from wildtype or Myd88 knockout (*KO*) mice were either stimulated with 10 μg/ml LPS + brefeldin A + monensin for 5 h. Intracellular IL-10 in B cells and macrophages were assessed by flow cytometry. Representative plots are shown in the *left panels*. The data shown in the right panels are the average percentages of IL-10^+^ cells ± standard deviation of biological replicates (*n* = 3 for wildtype, *n* = 5 for Myd88 knockout). ***, *p* < 0.001 (Student's two-tailed unpaired *t* test). *B*, peritoneal cavity cells from CD45.1+ve wildtype and CD45.2+ve Myd88 knockout mice were isolated, and equal numbers of cells from each genotype were mixed. The cells were then stimulated *ex vivo* with 10 μg/ml LPS + brefeldin A + monensin for 5 h. The cells were stained for CD45.1, CD45.2, CD19, and IL-10 and analyzed by flow cytometry. Representative plots for IL-10 in the B-cell population (CD19+ve) and non–B-cell population (CD19−ve) are shown, and the graphs represent the means ± standard deviation of four biological replicates. *, *p* < 0.05; **, *p* < 0.01 (Student's two-tailed unpaired *t* test).

In macrophages, LPS–dependent cytokine production can be inhibited by knockout of the Myd88 signaling adaptor. In line with this, Myd88 knockout reduced IL-10 production in peritoneal B cells (Fig. 2*A*). To confirm that the role of Myd88 was cell intrinsic in the B cells, LPS stimulations were carried out *ex vivo* on a mixed culture of peritoneal cells from CD45.2+ve Myd88 knockout mice and CD45.1+ve wildtype mice. This confirmed that loss of Myd88 specifically in the B cells could block LPS–induced IL-10 production (Fig. 2*B*).

### MAPK signaling is activated downstream of TLR activation in B cells and is required for IL-10 production

**Figure 3.**
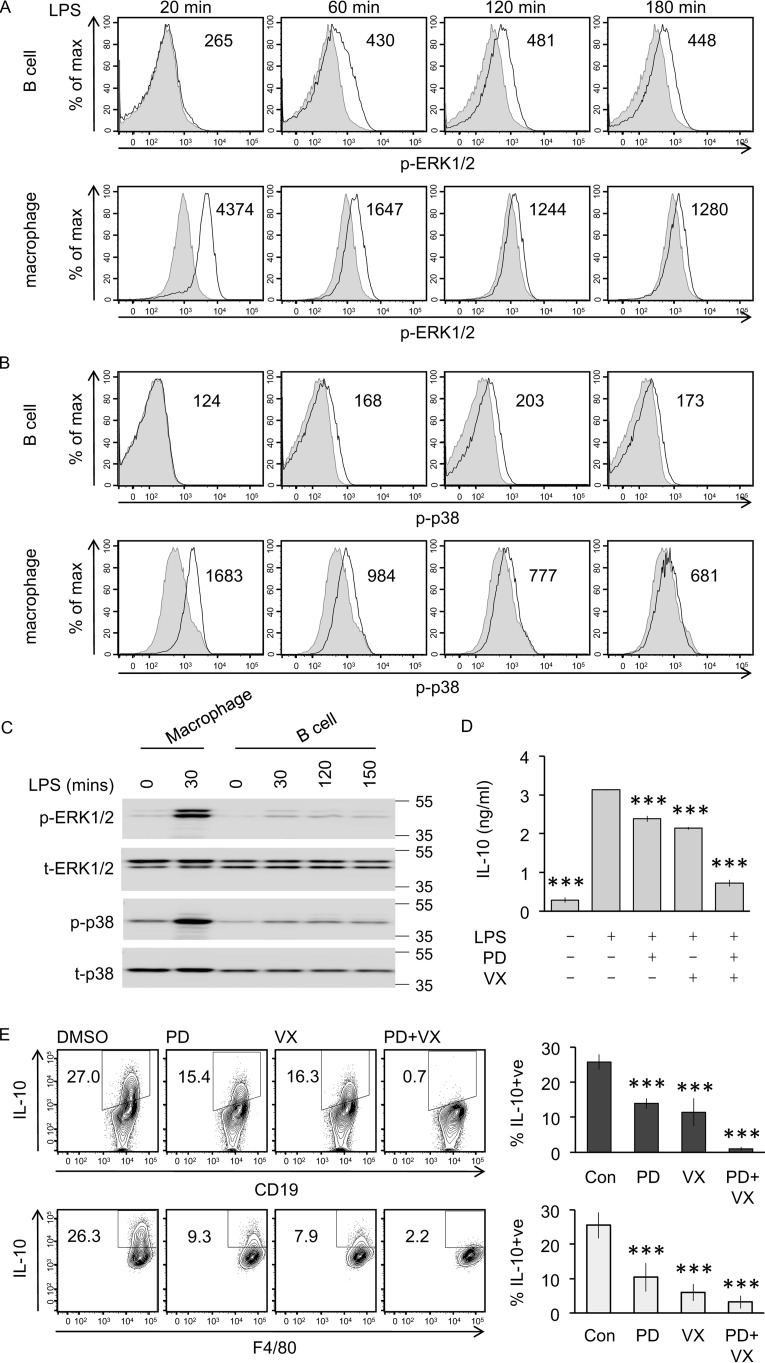
**ERK1/2 and p38 regulate LPS–induced IL-10 production.** *A* and *B*, peritoneal cavity cells from wildtype mice were stimulated with 10 μg/ml LPS for the indicated times and were stained for p-ERK1/2 (*A*), p-p38 (*B*), and CD19 and F4/80 lineage markers. Representative plots are shown for the p-ERK1/2 and p-p38 signals in the CD19 B-cell and F4/80 macrophage population (*black line*) overlaid on the basal signal in unstimulated controls (*gray*, *filled*). The results are representative of three independent experiments. *C*, peritoneal cavity cells from wildtype mice were FACS sorted into pure B-cell and macrophage populations as described under “Materials and methods” before stimulation with 10 μg/ml LPS for the indicated times. Phospho- and total levels of ERK1/2 and p-p38 were assessed by immunoblotting. *D*, peritoneal cavity cells from wildtype mice were cultured at 2 × 10^6^ cells/ml. The cells were either incubated with 2 μm PD284352 (*PD*), 1 μm VX-745 (*VX*) as indicated or with 0.1% DMSO for 1 h prior to stimulation with 10 μg/ml LPS for 16 h. Culture supernatant was harvested, and concentration of secreted IL-10 was determined by a Luminex assay. The data shown are means ± standard deviation of biological quadruplicates (*n* = 4). One-way ANOVA for the effect of inhibitor treatment on the IL-10 concentration was significant (*F*(4,15) = 1514, *p* < 0.001). For pairwise comparisons with LPS–stimulated cells without inhibitor using the Holm–Sidak method, *** indicates *p* < 0.001. *E*, peritoneal cavity cells from wildtype mice were either incubated with 2 μm PD284352, 1 μm VX-745 as indicated or with 0.1% DMSO for 1 h prior to stimulation with 10 μg/ml LPS + brefeldin A + monensin for 5 h. Intracellular IL-10 in B cells and macrophages was assessed by flow cytometry. Representative plots are shown in the *left panel*, and the graphs in the *right-hand panel* represent the averages and standard deviation of biological replicates (*n* = 4). One-way ANOVA for the effect of inhibitor treatment on the percentage of IL-10–positive cells was significant in B cells (*F*(3,12) = 72.037, *p* < 0.001) and macrophages (*F*(3,12) = 38.8, *p* < 0.001). For pairwise comparisons with LPS–stimulated cells without inhibitor using the Holm–Sidak method, *** indicates *p* < 0.001. *Con*, control.

LPS activates a number of signaling cascades, of which the ERK1/2 and p38 play a central role in IL-10 induction in macrophages (20). We therefore looked at the ability of LPS to activate these pathways in the peritoneal B cell and macrophage subsets. LPS–stimulated cells were stained intracellularly using fluorophore-labeled phospho-ERK1/2 (p-ERK1/2) and phospho-p38 (p-p38) antibodies that recognize these kinases when they are phosphorylated on their T*X*Y activation motifs. Strong phosphorylation of ERK1/2 (Fig. 3*A*) and p38 (Fig. 3*B*) was seen in macrophages by 20 min of LPS stimulation, which then decreased over time. In B cells, the response was slower with little or no ERK1/2 phosphorylation seen at 20 min; however, p-ERK1/2 was seen at 60 min, which was sustained and still apparent at 180 min (Fig. 3*A*). Like ERK1/2, p38 phosphorylation in B cells was both delayed and weaker compared with that observed in macrophages (Fig. 3*B*). To confirm these results, peritoneal cells were sorted into purified populations of B cells and macrophages by FACS prior to LPS stimulation, and immunoblotting was used to assess ERK1/2 and p38 phosphorylation. Corroborating the flow cytometry data, ERK1/2 and p38 phosphorylation was seen in LPS–stimulated purified B cells; however, phosphorylation of these kinases was weaker and delayed compared with that seen in macrophages (Fig. 3*C*).

Treatment of peritoneal cells before LPS stimulation with the MKK1/2 inhibitor PD184352, which blocks the activation of ERK1/2, reduced the secretion of IL-10 (Fig. 3*D*). The p38α/β inhibitor VX-745 resulted in a similar reduction in IL-10 production. ERK1/2 and p38α are known to have some common substrates in cells including MSK1 and 2 (20), and a combination of PD184352 and VX-745 resulted in stronger inhibition of IL-10 production than either inhibitor on its own (Fig. 3*D*). Measurement of cytokine levels present in the media from mixed cell cultures does not distinguish between cytokine secretion by the B-cells and macrophage subsets. To determine whether the ERK1/2 and p38α pathways regulate IL-10 production in B cells and macrophages, the experiments were repeated using intracellular IL-10 staining as the readout. Treatment of peritoneal cells with PD184352 or VX-745 prior to LPS stimulation reduced IL-10 induction in B cells, whereas a combination of both PD184352 and VX-745 blocked IL-10 production (Fig. 3*E*). PD184352 and VX-745 also reduced IL-10 production in peritoneal macrophages (Fig. 3*E*), consistent with what has been published in bone marrow–derived macrophages (22, 27).

### TLR–induced IL-10 production in B cells is independent of MSK1/2 and CREB activation

In bone marrow–derived macrophages, LPS–induced IL-10 production has previously been shown to be dependent on the kinases MSK1 and 2, which act downstream of both the ERK1/2 and p38 MAPK pathways. In these cells, MSKs act via phosphorylating the transcription factor CREB to promote the transcription of CREB dependent genes (27, 28).

**Figure 4.**
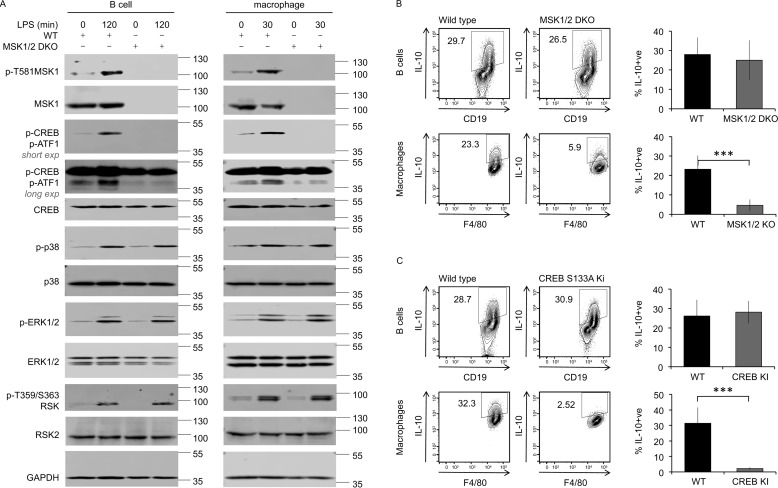
**MSK and CREB regulate LPS–induced IL-10 production in macrophages but not B cells.** *A*, B-cell and macrophage populations were isolated from peritoneal cavity cells from wildtype and MSK1/2 double knockout mice. The cells were treated for the indicated times with 10 μg/ml LPS or left unstimulated. The levels of the indicated proteins were assessed in cell lysates by immunoblotting. Long and short exposures of the phosphor-CREB/ATF1 blot are shown to allow visualization of both phospho-CREB (*upper band*) and phospho-ATF1 (*lower band*). *B* and *C*, peritoneal cavity cells from wildtype, MSK1/2 double knockout (*B*), and CREB S133A knockin (*C*) mice were stimulated with 10 μg/ml LPS + brefeldin A + monensin for 5 h. Intracellular IL-10 in B cells and macrophages was assessed by flow cytometry. Representative plots are shown in the *left panels*. The data shown in the *right panels* represent the averages and standard deviations of biological replicates (*n* = 12 in *B*, *n* = 6 in *C*). ***, *p* < 0.001 (two-tailed unpaired Student's *t* test).

To examine MSK activation in B cells, B cells and macrophages were purified from the peritoneal cavity cells via magnetic sorting, as described under “Materials and methods.” MSK1 activation requires it to be phosphorylated on Thr^581^ by the upstream MAPK (41). LPS stimulation induced Thr^581^ phosphorylation of MSK1 in both peritoneal B cells and macrophages from wildtype mice (Fig. 4*A*). LPS also induced the phosphorylation of CREB and the related transcription factor ATF1 in wildtype, but not MSK1/2 knockout, B cells (Fig. 4*A*). As expected, CREB phosphorylation following LPS stimulation in isolated macrophages was also MSK–dependent (Fig. 4*A*). Similar results were also obtained from B cells isolated by FACS (data not shown). These data show that LPS stimulation activated the MSK1/2–CREB pathway in peritoneal B cells, similar to what was observed in peritoneal macrophages (Fig. 4*A*).

**Figure 5.**
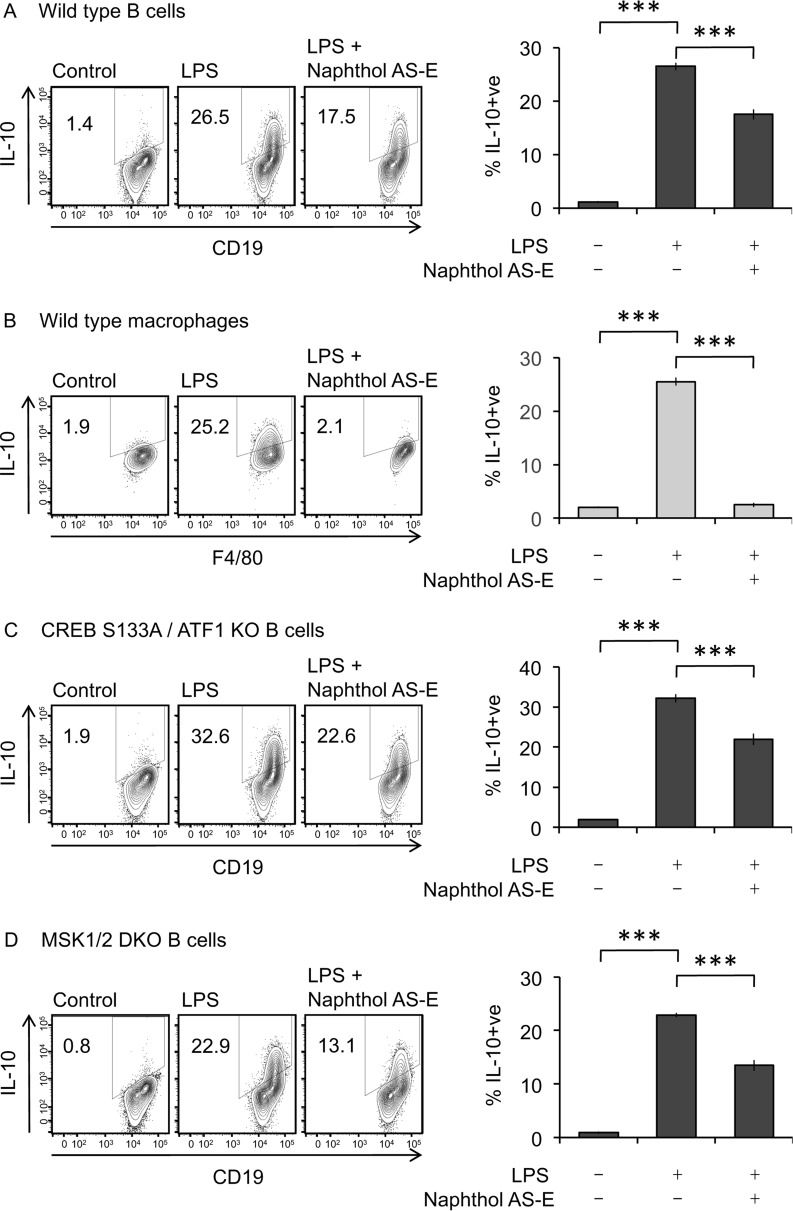
**Naphthol AS-E inhibits IL-10 induction in B cells independently of CREB phosphorylation.** *A* and *B*, wildtype peritoneal cells were incubated with 20 μm Naphthol AS-E for 1 h. The cells were then either stimulated with 10 μg/ml LPS + brefeldin A + monensin for 5 h or treated with brefeldin A + monensin alone. *A* and *B*, intracellular IL-10 in B cells (*A*) and macrophages (*B*) was assessed by flow cytometry. Representative plots are shown in the *left panels*. The data shown in the *right panels* represent the averages ± standard deviations of three stimulations. ***, *p* < 0.001 relative to the LPS–stimulated cells (two-tailed unpaired Student's *t* test). *C*, as in *A* except peritoneal cells were isolated from CREB S133A/ATF1 KO mice. *D*, as in *A* except peritoneal cells were isolated from MSK1/2 double knockout mice.

Next, the role of MSK and CREB in LPS–stimulated IL-10 induction in peritoneal cells was examined. Intracellular IL-10 staining in peritoneal cells from MSK1/2 knockout or CREB S133A knockin mice (in which the MSK phosphorylation site in CREB, Ser^133^, has been mutated to alanine) was compared with staining in peritoneal cells from matched wildtype control mice. Consistent with previous studies in BMDMs (27, 28), MSK1 and MSK2 double knockout (Fig. 4*B*) or S133A mutation of CREB (Fig. 4*C*) reduced IL-10 induction by peritoneal macrophages. Unexpectedly, and in contrast to the results in macrophages, in the same experiments neither MSK1/2 knockout nor the CREB S133A knockin had any effect on the ability of peritoneal B cells to produce IL-10 in response to LPS (Fig. 4, *B* and *C*). Recently IL-10 induction in B cells has been reported to be reduced by Naphthol AS-E (42), a compound that can inhibit the interaction of CREB with its co-activator protein CBP (43, 44). The interaction of CREB and CBP required CREB to be phosphorylated on Ser^133^, and it has previously been shown that CBP is not recruited to CREB in cells from the S133A knockin mice. We therefore tested the effect of Naphthol AS-E on IL-10 induction. In wildtype peritoneal cells, Naphthol AS-E reduced IL-10 induction in B cells in response to LPS and blocked IL-10 induction in macrophages (Fig. 5, *A* and *B*). To test whether this inhibition was due to an on target effect of the inhibitor, peritoneal cells were isolated from mice with a S133A knockin mutation in CREB (which blocks CBP recruitment) and a knockout of the CREB–related transcription factor ATF1. Naphthol AS-E reduced IL-10 induction in the B cells from these mice to a similar extent as seen in wildtype cells (Fig. 5*C*), suggesting that the effect of this compound was not acting via CREB. In line with this, Naphthol AS-E also reduced IL-10 induction in MSK1/2 knockout cells (Fig. 5*D*). Together, these data show that, unlike macrophages, B-cell IL-10 production is not regulated by MSK1/2 or CREB phosphorylation.

### RSK and MK2/3 are required for maximal TLR-induced IL-10 production in B cells

The above data show that ERK1/2 and p38 MAPK signaling regulate TLR–induced IL-10 production in B cells, but that unlike in macrophages this does not require MSK1 and 2 downstream of MAPK activation. We therefore assessed the potential role of other ERK1/2 and p38 MAPK–activated kinases in B-cell IL-10 production.

**Figure 6.**
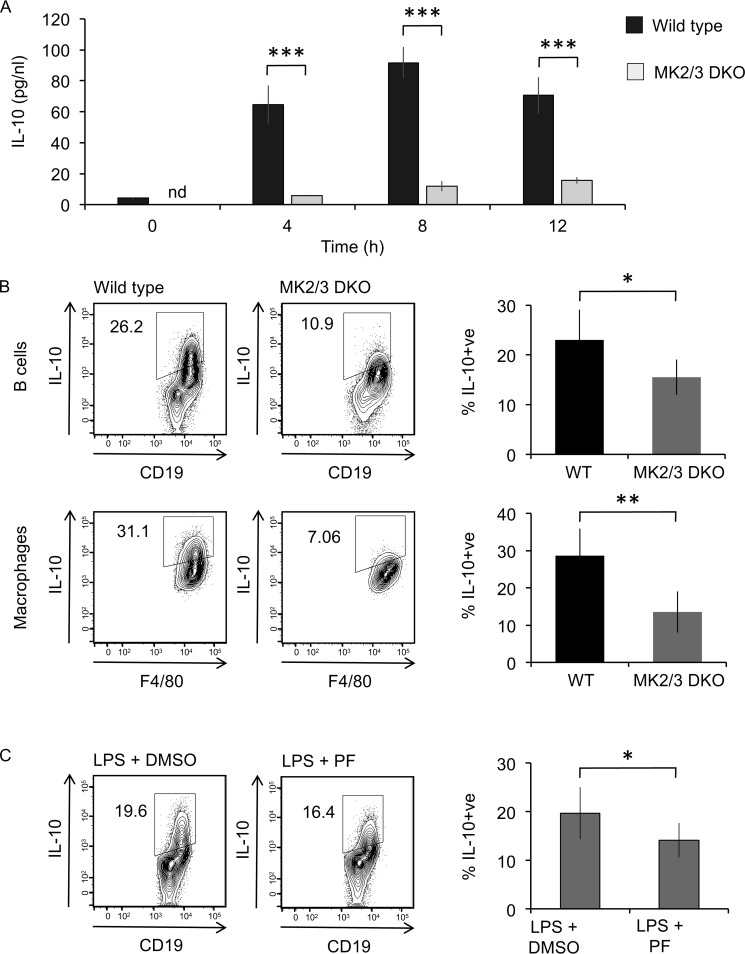
**MK2 and 3 contribute to LPS–induced IL-10 production.** *A*, BMDMs were cultured from wildtype and MK2/3 double knockout (*DKO*) mice. The cells were stimulated with 100 ng/ml LPS for the indicated times, and the levels of IL-10 in the culture supernatant were determined using a Luminex assay. The data show the means ± standard deviation from cultures from four mice/genotype. *B*, peritoneal cavity cells from wildtype and MK2/3 double knockout mice were stimulated with 10 μg/ml LPS + brefeldin A + monensin for 5 h. Intracellular IL-10 in B cells and macrophages was assessed by flow cytometry. Representative plots are shown in the *left panels*. The data shown in the *right panels* represent the averages ± standard deviations of biological replicates (*n* = 4). *C*, peritoneal cavity cells from wildtype mice were incubated with 5 μm PF3644022 for 1 h prior to stimulation with 10 μg/ml LPS + brefeldin A + monensin for 5 h. Intracellular IL-10 levels in B cells was assessed by flow cytometry. Representative plots are shown in the *left panels*. The data shown in the *right panel* represent the averages ± standard deviations of biological replicates (*n* = 7). *, *p* < 0.05; **, *p* < 0.01; ***, *p* < 0.001 compared with LPS–stimulated WT cells (two-tailed unpaired Student's *t* test).

The p38α-activated kinases MK2 and MK3 have previously been implicated in regulating the induction of several cytokines, including IL-10, in macrophages (25). In agreement with this MK2/3 double knockout BMDMs secreted significantly less IL-10 than wildtype BMDMs, in response to LPS stimulation (Fig. 6*A*). Similarly, on LPS stimulation peritoneal cavity macrophages from MK2/3 double knockout mice showed reduced IL-10 induction compared with macrophages from wildtype mice (Fig. 6*B*). Peritoneal cavity B cells also showed reduced IL-10 induction, but the decrease was less pronounced than that in macrophages (Fig. 6*B*). In agreement with this, inhibition of MK2/3 in wildtype cells with the MK2/3 inhibitor PF3644022 also had an inhibitory effect on IL-10 induction in B cells (Fig. 6*C*).

**Figure 7.**
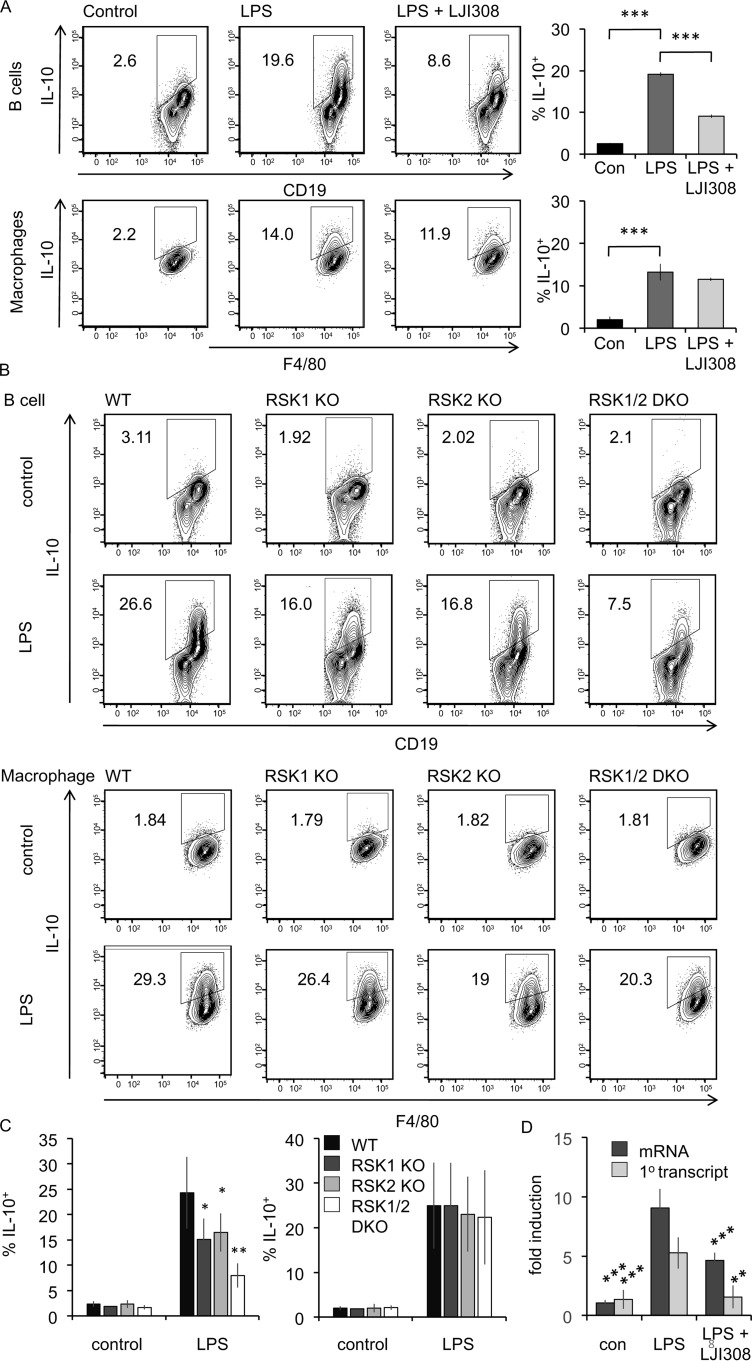
**RSK regulated IL-10 production in LPS–stimulated B cells.** *A*, peritoneal cavity cells from wildtype mice were incubated with 10 μm LJI308 for 1 h prior to stimulation with 10 μg/ml LPS + brefeldin A + monensin for 5 h. Intracellular IL-10 in B cells and macrophages was assessed by flow cytometry. Representative plots are shown in the *left panels*. The data shown in the *right panel* represent the averages ± standard deviations of biological replicates (*n* = 4). ***, *p* value < 0.001 compared with LPS–stimulated cells (two-tailed unpaired Student's *t* test). *B*, peritoneal cavity cells from wildtype, RSK1 knockout (*KO*), RSK2 knockout, and RSK1/2 double knockout (*DKO*) mice were stimulated with 10 μg/ml LPS + brefeldin A + monensin for 5 h. Intracellular IL-10 in B cells and macrophages was assessed by flow cytometry. Representative plots are shown. *C*, quantification of data from *B*. The graphs represent the averages ± standard deviations of biological replicates (*n* = 13, 5, 8, and 4 for LPS–stimulated WT, RSK1, RSK2, and RSK1/2, respectively). One-way ANOVA for LPS–treated samples showed a that the effect of genotype on the percentage of IL-10–positive cells was significant in B cells (*F*(3,26) = 10.745, *p* < 0.001) but not macrophages (*F*(3,26) = 0.128, *p* = 0.943). For pairwise comparisons with wildtype cells using the Holm–Sidak method, * indicates *p* < 0.05, and ** indicates *p* < 0.001. *D*, B cells were purified form peritoneal washes from wildtype mice as described under “Materials and methods.” The cells were incubated with 10 μm LJI308 for 1 h prior to stimulation with 10 μg/ml LPS for 2h. Total RNA was isolated, and induction of IL-10 mRNA and IL-10 primary (1^0^) transcript was determined by qPCR relative to unstimulated cells. Graphs show means ± standard deviations (*n* = 5). **, *p* < 0.01; ** *p* < 0.001 compared with LPS–stimulated cells (two-tailed Student's *t* test). *Con*, control.

Next, we explored the role of kinases downstream of ERK1/2 signaling in regulating B-cell IL-10 production. The RSK family of kinases comprises four members in mammalian cells (RSK1, 2, 3, and 4) and is related to MSK1 and 2. RSK1, 2, and 3 are activated in cells by ERK1/2, whereas RSK4 has been suggested to be constitutively active (reviewed in Refs. 23 and 45). RSK was activated in B cells by LPS, as judged by phosphorylation on Thr^359^/Ser^363^, sites which are phosphorylated by ERK1/2 and correlate with RSK activation (46). To test the requirement of RSK signaling for B-cell IL-10 production, experiments were carried out using both RSK inhibitors and RSK knockout mice. LJI308 has recently been reported to be a specific RSK inhibitor (47, 48). To determine the working concentration of LJI308 and to assess its specificity, LJI308 was titrated on PMA–stimulated HeLa cells (Fig. S2*A*). In these cells, PMA activates ERK1/2, which in turn activates RSK to promote GSK3 phosphorylation and MSK1/2 to promote CREB phosphorylation (30, 49). GSK3α/β can also be phosphorylated by Akt (49); therefore, these experiments were done in the presence of the PI3K inhibitor GDC0941 to block the PI3K–Akt pathway. A concentration of 10 μm of LJI308 could block GSK3α/β phosphorylation without affecting CREB phosphorylation, confirming that LJI308 was selective for RSK over MSK in cells (Fig. S2*A*). Furthermore *in vitro* screening of LJI308 against a panel of 140 kinases at 0.1 μm showed that only RSK1 and RSK2 were inhibited by more than 80%. RSK3 and RSK4 were not in the panel, and LJI308 did not show inhibition of MSK1, MK2, or MK3 in this assay (Fig. S2*D*). Assessment of IL-10 induction by intracellular staining in LPS–stimulated wildtype peritoneal cavity cells showed that LJI308 inhibited IL-10 induction in the B-cell subset but not in macrophages (Fig. 7*A*). Similar experiments were done with two other inhibitors: SB747651A, which has previously been shown to inhibit RSK in addition to MSKs (50); and Cmp-20, which has been reported as a dual RSK/MSK inhibitor (51). Titration of these inhibitors on PMA stimulated HeLa cells showed that either 10 μm Cmp-20 (Fig. S2*B*) or 10 μm SB747651A (Fig. S2*C*) was sufficient to inhibit both RSK and MSK, as assessed by immunoblotting for GSK3α/β and CREB phosphorylation, respectively. Both SB747651A and Cmp-20 inhibited LPS–induced IL-10 production in peritoneal B cells form wildtype mice (Fig. S3*A*). Because these compounds target both MSK and RSK, this experiment was repeated in cells from MSK1/2 knockout mice where any effect of the inhibitor would be independent of MSKs. Again, both SB747651A and Cmp-20 inhibited IL-10 production in peritoneal B cells from MSK1/2 knockout mice (Fig. S3*B*). Taken together, these data are consistent with a role for RSK in regulating B-cell IL-10 production.

Four isoforms of RSK have been described, of which RSK1, RSK2, and RSK3 are activated by the ERK1/2 pathway (23, 45). Interrogation of the ImmGen database indicated that the mRNA levels for RSK1 and RSK2 were higher than the levels for RSK3 and RSK4 in peritoneal B cells (Fig. S3*C*). To examine the role that RSK1 and RSK2 might play in IL-10 regulation in B cells, peritoneal cells were isolated from RSK1, RSK2, and RSK1/2 knockout mice. Double knockout of both RSK1 and RSK2 reduced LPS–stimulated IL-10 production relative to wildtype cells (Fig. 7, *B* and *C*). Single knockout of either RSK1 or RSK2 resulted in an intermediate effect, suggesting that there is compensation between RSK1 and RSK2 (Fig. 7, *B* and *C*). RSK1/2 knockout did not affect LPS–induced IL-10 production in peritoneal macrophages (Fig. 7, *B* and *C*). IL-10 production can potentially be regulated at multiple stages. To determine whether IL-10 could be regulated by RSK at the level of mRNA induction, RNA was isolated from peritoneal B cell stimulated in the presence or absence of the RSK inhibitor LJI308. This showed that RSK inhibition decreased the induction of IL-10 mRNA in response to LPS (Fig. 7*D*). mRNA levels can be affected by both changes in transcription and mRNA stability. Because the levels of the primary unspliced transcript of a gene can give a better indication of changes in transcription than mRNA levels, this was also analyzed for IL-10. As for the mRNA, the induction of the primary transcript for IL-10 in response to LPS was reduced by LJI308 (Fig. 7*D*). Overall, data from RSK inhibitor and RSK knockout mice experiments support a central role for RSK signaling in the regulation IL-10 production in B cells.

## Discussion

The ability of certain B-cell subsets to make IL-10 has emerged as an important control mechanism in the immune system (reviewed in Refs. 36 and 37). The majority of these studies have focused on the development and function of IL-10–producing B cells, rather than on the intracellular signaling that controls IL-10 production, which has remained largely uncharacterized. Knockout of the Ca^2+^ sensors STIM1 and STIM2 inhibits B-cell IL-10 production, suggesting a role for Ca^2+^ in the development or function of IL-10–producing B cells (52). Downstream of Ca^2+^, a role for NFAT has been proposed based on the use of calcineurin inhibitors (52, 53), although another study found the effect of calcineurin inhibitors on IL-10 was independent of NFAT (54). CD40 ligation in human tonsillar B cells has been found to induce IL-10 mRNA, and this was blocked by a p38 inhibitor (55). p38α/β and MEK1/2 inhibitors have also been found to reduce LPS–induced IL-10 secretion in murine splenic cultures enriched for B cells (56). In line with this, we find that LPS–induced IL-10 production in peritoneal B cells was reduced by inhibitors of the ERK1/2 and p38 MAPK pathways and that combining inhibitors of both pathways had an additive effect.

The molecular mechanism of TLR–induced IL-10 production has been more extensively studied in macrophages. In these cells, ERK1/2 and p38 MAPK pathways converge on the activation of MSK1 and 2. MSKs phosphorylate CREB, thereby activating its ability to drive IL-10 transcription (22, 27, 57–62). The studies reported here support the role for MSK and CREB in TLR–induced IL-10 production in macrophages. In contrast, they show that in peritoneal B cells, TLRs regulate IL-10 independently of MSKs and CREB, even though MSK catalyzed CREB phosphorylation occurs in B cells. A role for CREB regulating IL-10 in peritoneal B cells has recently been suggested based on the use of CREB–CBP interaction inhibitor Naphthol AS-E (42). Similar to Aziz *et al.* (42), we also found that Naphthol AS-E reduced IL-10 induction in wildtype B cells. This compound is not a direct CREB inhibitor; instead it binds to the KIX domain in CBP and prevents the interaction of the CBP KIX domain with the phosphorylated KID domain of CREB (43, 44). As a result, it prevents CBP recruitment to CREB and reduces the activation of CREB-dependent genes. Phosphorylation of Ser^133^ in the KID domain of CREB is also required for CBP recruitment (30), and therefore the use of Naphthol AS-E in the S133A knockin cells can distinguish between CREB–dependent and –independent effects of the compound. Because Naphthol AS-E reduced IL-10 induction in CREB S133A knockin B cells, the effects of Naphthol AS-E in this case are likely to be CREB–independent. The mechanism of action of Naphthol AS-E in B-cell IL-10 regulation is unclear. CBP can act as a co-activator for a large number of transcription factors, and for some of these, such as c-Myb, p53, and FOXO3a, CBP is recruited via interactions with its KIX domain (reviewed in Refs. 63 and 64). It is not known whether the binding of Naphthol AS-E to the CBP KIX domain blocks the recruitment of CBP to these transcriptions factors. It is, however, possible that the effect of Naphthol AS-E on IL-10 is due to it blocking CBP binding to a transcription factor distinct from CREB. Alternatively, it may be that Naphthol AS-E exerts effects in cells independent of CBP.

Our studies indicate that the control of IL-10 in B cells is independent of CREB and MSKs. Instead the ERK1/2–activated kinases RSK1 and RSK2 regulated IL-10 production in peritoneal B cells but not macrophages. A role for ERK1/2 in IL-10 induction has been observed in macrophages stimulated with *Schistosoma mansoni* excretory products, which was suggested to be RSK–dependent (65). Although this study did find evidence of RSK phosphorylation in macrophages, it did not directly look at the effect of RSK inhibition on IL-10 induction or CREB phosphorylation, and the data presented in the study on p38 and ERK1/2 inhibition on IL-10 fits with the canonical MSK–CREB dependent pathway in macrophages. Of note, we show here that neither RSK1/2 knockout nor a selective RSK inhibitor blocked LPS–induced IL-10 production in macrophages. Taken together, the results indicate that macrophages and B cells use distinct mechanisms to regulate IL-10 downstream of ERK1/2 and highlights that immune cell–specific differences in signaling occur downstream of a common receptor.

## Materials and methods

### Reagents

DMEM, PBS, RPMI, l-glutamine, and penicillin-streptomycin were from Life Technologies (Invitrogen); EDTA was from Ambion; 2-mercaptoethanol, DMSO, and BSA were from Sigma; non-essential amino acids, sodium pyruvate, and HEPES buffer were from Lonza; brefeldin A and monensin were from eBiosciences; fetal calf serum (FCS) was from Labtech. LPS from *Escherichia coli* 026:B6 was from Sigma. CL-097 (TLR7/8 agonist), Pam3CSK4 (TLR1/2 agonist), and CpG (ODN1826, TLR9 agonist) were from InvivoGen.

### Antibodies

Flow cytometry antibodies to CD19 (clone eBio1D3), IL-10 (clone JEs5-16E3), TNFα (clone MP6-XT22), IgM (clone 11/41), and IgD (clone 11-26c.2a) were from eBiosciences; F4/80 (clone BM8), CD1d (clone 1B1), and CD5 (clone 53-7.3) were from BioLegend; IL-6 (clone MP5-20F3) was from BD Biosciences; anti-rabbit IgG Fab2–PE conjugate was from Cell Signaling Technology; and rat anti-mouse CD16/CD32 Fc block^TM^ (clone 2.4G2) was from BD Pharmingen^TM^. Western blotting antibodies to total MSK1 and total RSK2 were made in-house. Phospho-ERK1/2 (Thr^202^/Tyr^204^) (catalog no. 9101), total ERK1/2 (catalog no. 9102), phospho-p38 (Thr^180^/Tyr^182^) (catalog no. 4511), total p38 (catalog no. 9212), phospho-CREB (Ser^133^)/phospho-ATF1 (Ser^63^) (catalog no. 9198), total CREB (catalog no. 9197), phospho-MSK1 (Thr^581^) (catalog no. 9595), phospho-GSK3α/β (S21/S9) (catalog no. 9331), phospho-RSK (Thr^359^/Ser^363^ numbered for human RSK1) (catalog no. 9344), and total GAPDH (catalog no. 2118) were from Cell Signaling Technology.

### Inhibitors

The ERK1/2 inhibitor PD184352 was from Axon and was used at a final concentration of 2 μm. The p38 inhibitor VX-745 was from Selleck and was used at a final concentration of 1 μm. The PI3K inhibitor GDC-0941 was from Axon and was used at a final concentration of 1 μm. The MK2/3 inhibitor PF3644022 was from Tocris and was used at a final concentration of 5 μm. These concentrations were used because they have previously been established to inhibit the target kinases in cultured cells (50, 66, 67). The N-terminal MSK1/2 inhibitor SB747651A, which also inhibits RSK, was from Axon and was used at a final concentration of 10 μm. The C-terminal RSK/MSK1/2 inhibitor compound 20 (51) was generated in-house and was used at a final concentration of 10 μm. The RSK inhibitor LJI308 (47, 48) was also generated in-house and was used at a final concentration of 10 μm unless otherwise stated. The selectivity of PD184352, VX745, and SB747641 has been published previously (33, 50, 66). The selectivity of LJI308 is given in Fig. S2. The CREB–CBP interaction inhibitor Naphthol AS-E (CAS 92-78-4) was from Calbiochem and used at 20 μm.

### Mice

MSK1, MSK2, RSK1, RSK2, MK2, and MK3 knockout mice have been described previously (33, 68–72). To generate the CREB S133A mouse, animals with a conditional CREB S133A knockin (71) were crossed onto a Vav-Cre transgenic background (73) to generate the knockin in hematopoietic cells. Where indicated the CREB mice were crossed to an ATF1 S63A knockin line, in which the mutated exon was flanked by loxP sites. When crossed to the Vav-Cre transgene, this resulted in immune cells with an S133A mutation in CREB and knockout of AFT1. All mice were maintained on a C57Bl/6 background (>12 generations). The mice were housed in individually ventilated cages at 21 °C, 55–65% humidity, and a 14/10-h light/dark cycle. The mice were provided with free access to food (R&M3) and water and kept under specific pathogen-free conditions.

### Cell isolation and culture

Murine peritoneal cavity was flushed with peritoneal cavity wash buffer (PBS + 1% BSA + 2 mm EDTA) by carefully injecting in 5–10 ml of buffer, gently massaging the abdomen, and collecting/aspirating the buffer with a syringe. Isolated cells were cultured in RPMI medium supplemented with 4 mm l-glutamine, 100 units/ml penicillin, 100 μg/ml streptomycin, 10 mm HEPES buffer, 100 μm (1×) non-essential amino acids, 1 mm sodium pyruvate, 50 μm 2-mercaptoethanol, and 10% heat inactivated FCS (peritoneal cell culture medium). Where indicated, the cells were treated with inhibitors for 1 h before stimulation. The cells were stimulated with 10 μg/ml LPS in the presence of Golgi inhibitors, 3 μg/ml brefeldin A, and 2 μm monensin. The cells treated with 3 μg/ml brefeldin A and 2 μm monensin but without LPS stimulation were used as controls. The cells were incubated at 37 °C, 5% CO_2_ for 5 h, unless indicated otherwise.

Bone marrow–derived macrophages were isolated as previously described (74) and cultured in BMDM (DMEM supplemented with 10% heat-inactivated fetal bovine serum, 2 mm l-glutamine, 100 units/ml of penicillin G, 100 μg/ml of streptomycin, 0.25 μg/ml of amphotericin [Invitrogen], and 5 ng/ml of macrophage colony-stimulating factor; PreProTech). HeLa cells were cultured in DMEM supplemented with 10% FBS, 2 mm l-glutamine, 100 units/ml of penicillin G, and 100 μg/ml of streptomycin and serum-starved for 16 h before stimulation.

### Flow cytometry

#### 

##### Cytokine staining

The cells were isolated, cultured, and stimulated as above. Sequentially, the cells were washed with FACS buffer (PBS + 1% BSA), fixed using fixation buffer (eBiosciences) for 20 min at +4 °C, washed with FACS buffer, permeabilized with 1× permeabilization buffer (eBiosciences) for 15 min at +4 °C, washed with FACS buffer, incubated with 1:100 Fc block made up in 1× permeabilization buffer for 10 min at +4 °C, washed with FACS buffer, and incubated with antibodies (1:100 CD19, 1:200 F4/80 and 1:100 IL-10 made up in 1× permeabilization buffer for 30 min at +4 °C. (Note that CD19 and F4/80 antibodies recognize fixed antigen; therefore extracellular CD19 and F4/80 staining and intracellular cytokine staining could be performed in a single step after fixing and permeabilizing cells.) Stained cells were washed with FACS buffer, resuspended in FACS buffer, and acquired on a BD FACSCanto^TM^ II.

##### Phosphoprotein staining

The cells were stimulated with LPS in the absence of Golgi inhibitors for the indicated times. The cells were fixed by adding ice-cold fixation buffer (two times the volume of media) to cells in media and incubating for 20 min on ice. The cells were washed with cold FACS buffer and permeabilized with ice-cold 90% methanol/distilled water, added slowly while vortexing to prevent cell clumping, for 30 min on ice. The cells were washed twice with excess FACS buffer to get rid of the methanol before incubation first with 1:100 Fc block in FACS buffer and then with phospho-protein antibodies (1:50 p-ERK1/2 or 1:50 p-p38 Western blotting antibodies from Cell Signaling Technologies) in FACS buffer for 1 h at room temperature. The cells were washed twice with excess FACS buffer and incubated with 1:1000 anti-Rabbit IgG Fab–PE conjugate detection antibody + 1:100 CD19 + 1:200 F4/80 in FACS buffer for 30 min at room temperature in the dark. The cells were washed twice with excess FACS buffer, resuspended in FACS buffer, and acquired on a BD FACSCanto^TM^ II. All flow cytometry data were analyzed using FlowJo software.

### Fluorescence-activated cell sorting

The cells were isolated from the peritoneal cavity as above. Isolated cells from identical genotypes were pooled and stained for cell surface lineage markers, CD19 and F4/80, under sterile conditions and using 0.22-μm filter-sterilized reagents. Briefly, the cells were pelleted and incubated sequentially with 1:100 filter-sterilized Fc block made up in FACS buffer for 10 min at +4 °C and with filter-sterilized antibodies (1:100 CD19 and 1:200 F4/80) made up in FACS buffer for 30 min at +4 °C. The cells were washed with sterile FACS buffer, resuspended in sterile FACS buffer, and sorted into CD19^+^ and F4/80^+^ populations on a BD Influx^TM^ cell sorter into RPMI media containing 20% FCS. Sorted cells were pelleted and rested in peritoneal cell culture media for 2 h at 37 °C, 5% CO_2_ before use in assays.

### Western blotting

Peritoneal cavity cells were isolated as above. In some experiments, the cells were sorted into B cells (CD19^+^) and macrophages (F4/80^+^) as described above, before stimulation. All cells were rested for 2 h in media at 37 °C, 5% CO_2_ after isolation with or without inhibitors for the last 1 h, before stimulation as indicated in the figure legends. The cells were lysed in SDS lysis buffer (Triton lysis buffer is 50 mm Tris-HCl, pH 7.5, 1 mm EGTA, 1 mm EDTA, 1 mm sodium orthovanadate, 50 mm sodium fluoride, 1 mm sodium pyrophosphate, 10 mm sodium glycerophosphate, 0.27 m sucrose, 1% (v/v) Triton X-100. SDS lysis buffer is Triton lysis buffer mixed with 10% (v/v) glycerol, 1% (w/v) SDS, and bromphenol blue) supplemented with 14.3 mm 2-mercaptoethanol and protease inhibitors 1 μg/ml aprotinin, 1 μg/ml leupeptin, and 1 mm phenylmethylsulfonyl fluoride. The samples were syringed with a 25-gauge needle to shear DNA and heated at 100 °C for 10 min to denature proteins before running on a 10% SDS-polyacrylamide gel. Resolved proteins were transferred to a nitrocellulose membrane, and the membranes were probed for phospho- or total proteins as indicated. Images were acquired on an Odyssey® Fc Imaging system (Li-Cor Biosciences) and analyzed using Image Studio Lite software (Li-Cor Biosciences).

### Analysis of secreted cytokines

Sorted cells (B cells or macrophages) were cultured in 96-well plates at 1 × 10^5^ cells/well in 100 μl of media. The cells were stimulated with LPS in the absence of Golgi inhibitors, and culture supernatant was harvested at the indicated incubation times. Concentration of cytokines in the supernatant was assessed using a Luminex-based multiplex assay (Bio-Plex® System; Bio-Rad) according to the manufacturer's protocol.

### qPCR

B cells were isolated from total peritoneal cavity cells by positive selection using the magnetic antibody cell sorting separation system. The cells were stained with biotinylated anti-mouse CD19 (BioLegend) followed by incubation with streptavidin microbeads (Miltenyi Biotec), and B cells were purified on a magnetic antibody cell sorting separation column (Miltenyi Biotec). The purity of isolated B cells was routinely checked by flow cytometry and determined to be >95%. Following isolation cells were incubated in peritoneal cell culture media. For qPCR, following stimulation, total RNA was isolated from 1 × 10^6^ cells/sample using NucleoSpin RNA II purification kits (Macherey–Nagel) and 250 ng of RNA reverse transcribed using iScript (Bio-Rad). qPCR using SYBR Green–based detection (Takara Biosciences) was carried out as previously described (27, 75). Fold change was calculated relative to unstimulated cells and corrected for levels of 18s as a loading control. Primer sequences used were CCCTTTGCTATGGTGTCCTTTC and GATCTCCCTGGTTTCTCTTCCC for IL-10 mRNA, CCAAGCCTTATCGGAAATGA and TTTGTTGGGTGGCTCTAAGG for the primary IL-10 transcript, and GTAACCCGTTGAACCCCATT and CCATCCAATCGGTAGTAGCG for 18s.

### Graphs and Statistics

All of the graphs (mean ± S.D.) were plotted, and statistics analyses were performed using Microsoft Excel software. Two-tailed, unpaired Student's *t* test was carried out in Excel and one-way ANOVA using SigmaPlot 12.5.

## Author contributions

R. V. S., R. B., I. P., and A. M. carried out experiments and analyzed data; N. S., S. L., and A. W. synthesized RSK and MSK inhibitors; and J. S. C. A. and R. V. S. wrote the paper

## Supplementary Material

Supporting Information

## Footnotes

2 The abbreviations used are:

IL: interleukin
TLR: Toll-like receptor
MSK: mitogen- and stress-activated protein kinase
CREB: cAMP response element–binding protein
RSK: p90 ribosomal S6 kinase
LPS: lipopolysaccharide
BMDM: bone marrow–derived macrophage
p-: phospho-
PMA: phorbol 12-myristate 13-acetate
PI3K: phosphatidylinositol 3-kinase
MAPK: mitogen-activated protein kinase
MK: MAPK–activated protein kinase
DMEM: Dulbecco's modified Eagle's medium
FCS: fetal calf serum
qPCR: quantitative PCR
ANOVA: analysis of variance.
