# Supplementary material for: Differential control of Toll-like receptor 4–induced interleukin-10 induction in macrophages and B cells reveals a role for p90 ribosomal S6 kinases
Source: J Biol Chem. 2017 Dec 11;293(7):2302–17. doi: 10.1074/jbc.M117.805424 (PMC5818195; doi:10.1074/jbc.M117.805424)
Supplement: Supporting Information [file supp_293_7_2302__index.html]

Differential regulation of TLR4 induced IL-10 production in B cells and macrophages reveals a novel role for RSK1 and 2 in B cells. — Differential control of Toll-like receptor 4–induced interleukin-10 induction in macrophages and B cells reveals a role for p90 ribosomal S6 kinases — RSK1 and 2 regulate IL-10 induction in B cells — Supporting Information 

# Differential control of Toll-like receptor 4–induced interleukin-10 induction in macrophages and B cells reveals a role for p90 ribosomal S6 kinases

## Supporting Information

- supplemental figures (.pdf, 374 KB) - supplemental figures
