# Supplementary material for: Differential control of Toll-like receptor 4–induced interleukin-10 induction in macrophages and B cells reveals a role for p90 ribosomal S6 kinases
Source: J Biol Chem. 2017 Dec 11;293(7):2302–17. doi: 10.1074/jbc.M117.805424 (PMC5818195; doi:10.1074/jbc.M117.805424)
Supplement: Supporting Information [file 10.1074_M117.805424_jbc.M117.805424-1.pdf]

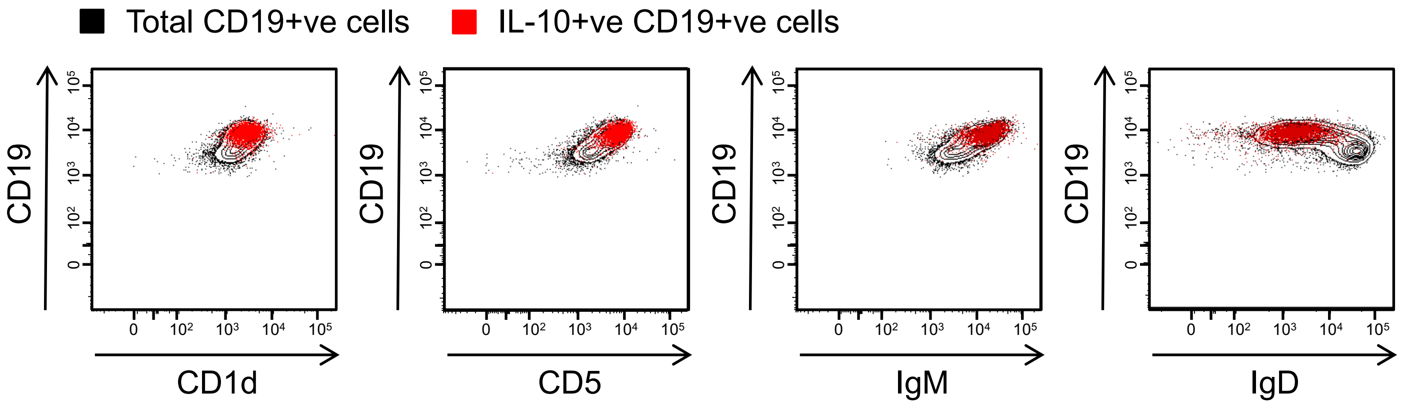

Supplementary Figure 1. Surface staining of cellular markers on total and IL-10+ve peritoneal B cells.

Peritoneal cavity cells were stimulated *ex vivo* with 10µg/ml LPS in the presence of 3µg/ml Brefeldin-A and 2µM Monensin for 5h. Cells were then fixed and stained for CD19, IL-10 and either CD1, CD5, IgM or IgD. Plots show the total live CD19+ve cell population in black and the IL-10 positive B cells in red.

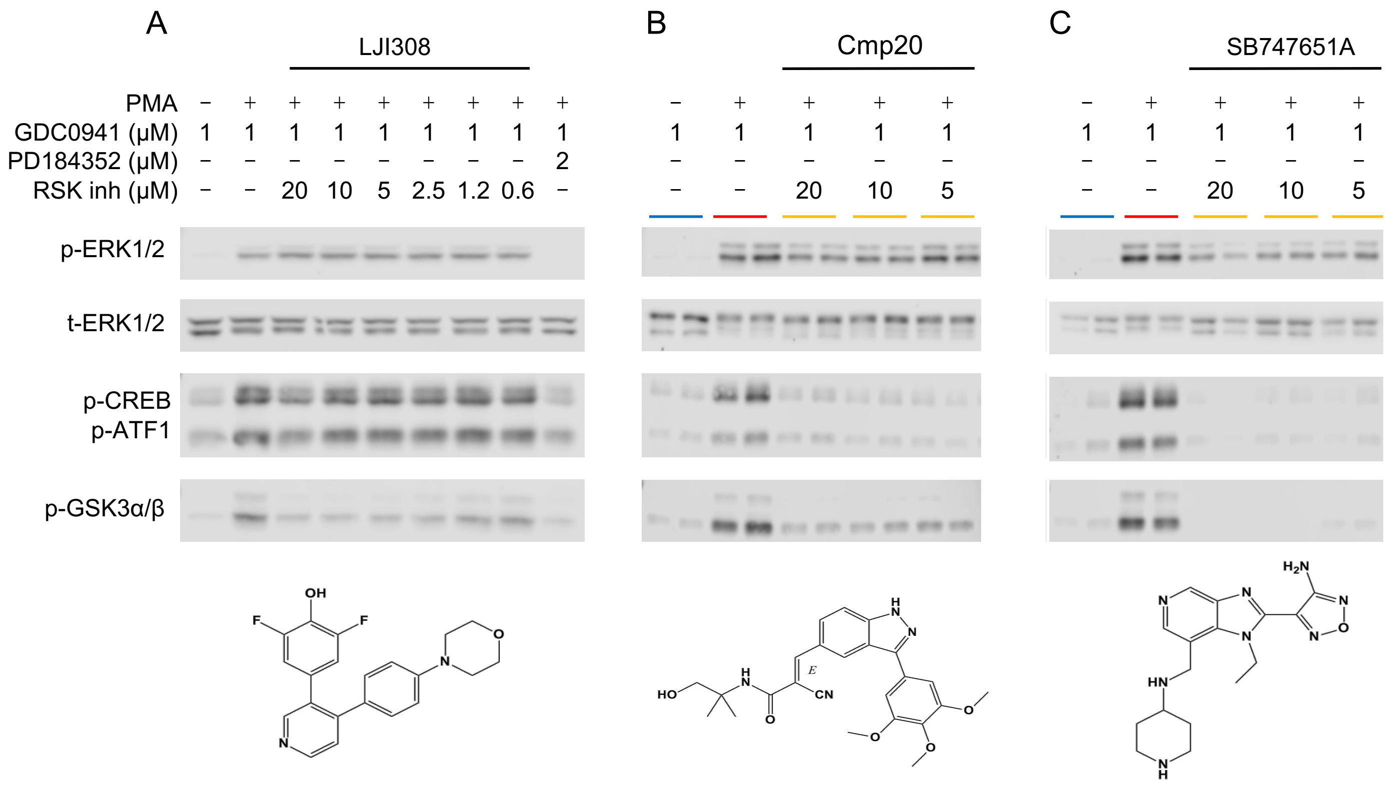

D

kinase	% activity remianing	variance	kinase	% activity remaining	variance	kinase	% activity remaining	variance	kinase	% activity remaining	variance
**RSK1**	**5**	1	JNK3	96	0	MNK2	103	24	GCK	110	13
**RSK2**	**15**	4	BRSK2	96	5	ULK2	103	7	MINK1	110	6
YES1	83	10	EF2K	96	0	NUAK1	103	12	SIK3	110	6
JNK2	84	6	FGF-R1	97	16	DYRK1A	104	4	p38b MAPK	110	1
VEG-FR	85	29	AMPK (hum)	97	6	PAK5	104	13	CDK2-Cyclin A	110	9
**MSK1**	**86**	14	CAMK1	98	23	CDK9-Cyclin T1	104	11	EPH-A4	111	13
TrkA	86	3	TIE2	98	11	Lck	104	3	MARK1	111	4
MARK2	87	3	MEKK1	98	15	ASK1	104	4	PKCγ	112	1
S6K1	88	2	PKCz	98	2	MAPKAP-K3	104	11	EPH-B2	112	4
HIPK2	88	25	RIPK2	98	6	Aurora A	104	19	PKA	112	0
ULK1	88	7	MLK3	98	13	PAK4	105	2	IRAK4	113	14
GSK3b	89	1	PKBa	98	15	ERK1	105	1	CSK	113	1
EPH-B4	89	4	MNK1	99	17	WNK1	105	9	TTBK2	114	17
DAPK1	90	1	OSR1	99	1	PRK2	105	2	Aurora B	114	9
HIPK1	91	0	PAK6	99	16	MST4	105	5	ERK8	114	4
MKK6	91	2	JAK3	99	3	MPSK1	106	1	BTK	114	5
p38d MAPK	91	3	TBK1	99	8	MKK1	106	20	MARK3	114	1
ABL	92	9	NEK2a	100	7	PKBb	106	17	PDGFRA	114	7
BRK	93	3	TLK1	100	4	PKD1	107	11	CLK2	115	18
PIM1	93	29	ROCK 2	100	7	LKB1	107	1	IKKe	116	11
TSSK1	93	2	DDR2	100	2	CK2	107	3	CHK2	116	7
ERK5	94	6	CK1δ	101	4	MLK1	107	1	ERK2	116	0
DYRK2	94	19	MARK4	101	11	DYRK3	107	0	PINK	116	20
CAMKKb	94	1	SYK	101	5	EPH-A2	107	25	MAP4K5	117	14
MKK2	94	11	IRAK1	101	14	STK33	108	6	TAO1	117	7
IKKb	94	8	PIM2	101	4	TTK	108	6	HER4	117	13
TESK1	95	4	PIM3	101	5	CK1γ2	108	6	SGK1	118	7
MST2	95	5	SIK2	102	6	PHK	108	7	IRR	118	3
MAPKAP-K2	95	3	BRSK1	102	3	EPH-B1	109	8	MST3	120	12
JNK1	95	5	PDK1	102	1	PAK2	109	8	TTBK1	120	8
SRPK1	95	5	TAK1	102	11	p38a MAPK	109	4	TGFBR1	121	4
ZAP70	96	3	PKCa	102	16	IR	109	8	p38g MAPK	123	6
MELK	96	8	PRAK	102	1	MAP4K3	110	16	PLK1	126	29
HIPK3	96	1	NEK6	102	4	EIF2AK3	110	2	EPH-B3	129	18
Src	96	8	SmMLCK	103	6	CHK1	110	15	IGF-1R	157	55

Supplementary Figure 2. MSK and RSK inhibitors

A – C HeLa cells were incubated with PI3K inhibitor 1 µM GDC0941 to inhibit Akt activation and block any Akt mediated GSK3 phosphorylation for 1h. Where indicated the cells were also incubated with the indicated concentrations of LJI308 (RSK inhibitor), Cmp20 (RSK/MSK inhibitor) or SB747651A (RSK/MSK inhibitor) for 1h. Cells were then stimulated in the presence of the inhibitors for 20min with 400ng/ml PMA to activate the ERK1/2 pathway. Cells were then lysed and the levels of total and phospho ERK1/2, phospho CREB and ATF1 (MSK substrate) and GSK3 (RSK substrate) determined by immunoblotting. (n=2). Structures from each compound are shown below the blots.

D RSKi-47 was screened against a panel of kinases in vitro. Data shows the % kinase activity remaining in the presence of 0.1 µM LJI308. Results show the average and variance of two assays.

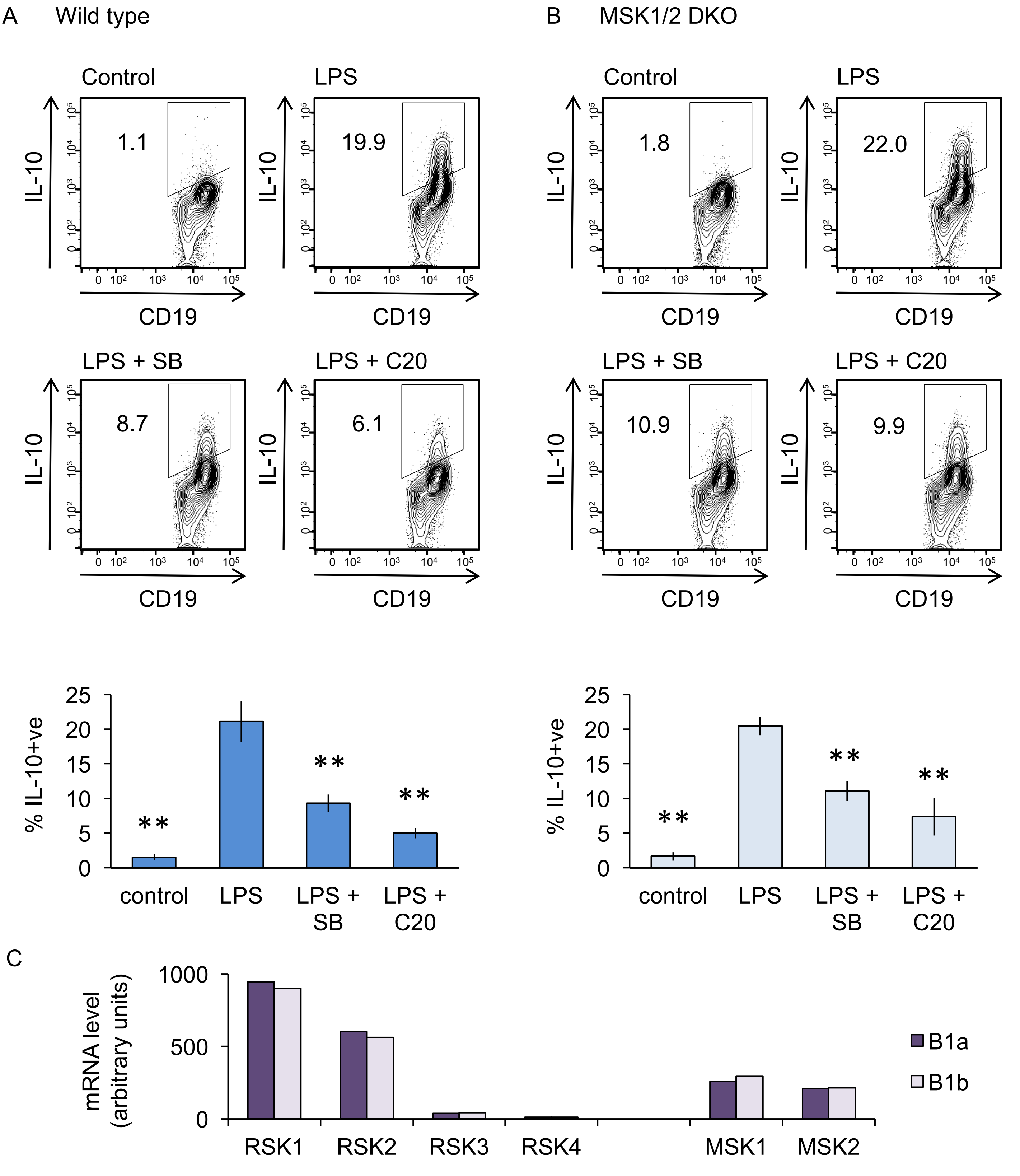

Supplementary figure 3. SB727651A and Compound-20 reduced LPS induced IL-10 production by peritoneal cavity B cells in wild type and MSK1/2 DKO mice.

A-B) Peritoneal cavity cells from wild type (A) and MSK1/2 double knock-out (B) mice were either incubated with MSK inhibitors 10 μM SB747651A (SB) or 10 μM Cmp-20 (C20), or with DMSO, for 1 h prior to stimulation with 10μg/ml LPS + Brefeldin A + Monensin for 5 h. Intracellular IL-10 in B cells (CD19+ve cells) was assessed by flow cytometry. Representative plots are shown in the left panels. Data shown in the right panels is average %IL-10^+ cells ± standard deviation of biological replicates (n = 4 for wild type, n = 3 for MSK1/2 double knock-out). One way ANOVA for treatment versus the % of IL-10 positive cells was significant in both wile type ($F(3,12)=267$, $p<0.001$) and MSK1/2 knockout ($F(3,8)=87.3$, $p<0.001$) cells. For pairwise comparisons (Holm-Sidak method) with the LPS condition a p value of less than 0.001 is indicated by**.

C) Relative mRNA levels for RSK isoforms in mouse peritoneal cavity B1 cells. (*Data from immunological genome project (www.immgen.org)).*
